# Identification, resistance mechanisms, and innovative therapeutic approaches against *Acinetobacter baumannii–calcoaceticus* complex

**DOI:** 10.3389/fmicb.2026.1869585

**Published:** 2026-07-08

**Authors:** Danish Daniyal, Cuiying Mao, Hongyan Shi

**Affiliations:** 1 Department of Pathogen Biology, College of Basic Medical Sciences, Jilin University, Changchun, China; 2 Department of Cardiology, China-Japan Union Hospital of Jilin University, Changchun, China

**Keywords:** *Acinetobacter baumannii–calcoaceticus* complex, antimicrobial resistance mechanisms, combination therapy, efflux pumps, horizontal gene transfer, molecular diagnostics, multidrug resistance, *β*-lactamases

## Abstract

*Acinetobacter baumannii–calcoaceticus* complex (ABC complex) is recognized as one of the most critical multidrug drugs resistant (MDR) pathogens worldwide and remains a major cause of hospital-acquired infections, particularly in intensive care settings. Members of this complex are associated with ventilator-associated pneumonia, bloodstream infections, wound infections, urinary tract infections, and meningitis, often affecting critically ill and immunocompromised patients. Their clinical importance is primarily driven by their remarkable ability to acquire, accumulate, and maintain resistance determinants against multiple classes of antimicrobial agents. The ABC complex acquires resistance through diverse and coordinated mechanisms, including the production of *β*-lactamases, target-site alterations, efflux pump overexpression, reduced membrane permeability, horizontal gene transfer (HGT), and the mobilization of insertion sequences and other genetic elements that modulate intrinsic and acquired resistance genes. The rapid dissemination of these determinants has significantly limited therapeutic options and contributed to global outbreaks. Accurate identification of individual members within the complex is essential, as closely related species may differ in epidemiology and resistance profiles. A comprehensive understanding of molecular resistance mechanisms, reliable diagnostic approaches, and evolving treatment strategies, including combination therapies and novel agents, is crucial. This review summarizes current knowledge on resistance mechanisms, identification methods, and innovative therapeutic strategies, highlighting the need for integrated clinical and microbiological efforts to combat ABC complex infections.

## 1 Introduction

The taxonomy of *Acinetobacter* has undergone substantial revision due to historical misclassification and progressive methodological advancements. Early isolates were variably assigned to genera such as *Diplococcus*, *Micrococcus*, *Alcaligenes*, *Mima*, *Moraxella*, *Herellea*, *Bacterium*, *Neisseria*, and *Achromobacter* based on shared phenotypic characteristics, including Gram-negative staining, non-motility, and lack of pigmentation. The genus *Acinetobacter* was formally proposed by Brisou and Prévot in 1954 in order to differentiate non-motile bacteria from motile species within the genus *Achromobacter*; however, early nomenclature remained inconsistent and taxonomically ambiguous. Members of the genus are Gram-negative, strictly aerobic, indole-negative, non-fastidious, nonmotile, catalase-positive, oxidase-negative, and citrate-positive bacteria, with a Deoxyribonucleic Acid (DNA) guanine–cytosine (G + C) content of 39–47% (Peleg et al., 2008). Continuous taxonomic refinement and the incorporation of molecular approaches have expanded the genus, and as of the Castanheira et al. (2023) (review), 74 species are recognized.

Within this genus, the ABC complex constitutes a clinically significant cluster whose classification has evolved with advances in molecular taxonomy. The complex includes *Acinetobacter baumannii (A. baumannii)*, *Acinetobacter nosocomialis (A.nosocomialis)*, *Acinetobacter pittii* (*A. pittii*), and *Acinetobacter calcoaceticus (A. calcoaceticus)*, as well as more recently described species such as *Acinetobacter seifertii (A.seifertii*) and *Acinetobacter djikshoorniae (A.djikshoorniae)* (Esen and Gözalan, 2020). Accurate species-level identification is essential because members differ in antimicrobial resistance (AMR) profiles, virulence potential, and clinical outcomes. Among these, *A. baumannii*is the most clinically significant and is frequently associated with MDR hospital-acquired infections (Marinova-Bulgaranova et al., 2025). *A. nosocomialis* is also implicated in nosocomial infections (Kamolvit, 2015). While *A. pittii* has emerged as a pathogen harboring notable resistance determinants (Castanheira et al., 2023). In contrast, *A. calcoaceticus* is primarily environmental and less commonly associated with human disease (Kamolvit, 2015).

The ABC complex represents a major global public health concern due to its extensive distribution, genetic adaptability, and high levels of AMR. In clinical settings, particularly intensive care units, it is a leading cause of healthcare-associated infections, including ventilator-associated pneumonia, bloodstream infections, and urinary tract infections. Global dissemination is largely driven by a limited number of high-risk MDR clones (Castanheira et al., 2023; Lysitsas et al., 2023). The burden of disease is exacerbated by resistance to multiple antibiotic classes, including carbapenems and colistin, mediated by horizontal gene transfer, plasmid- and integron-associated resistance genes such as beta-lactamase oxacillinase (*blaOXA)* and New Delhi metallo-beta-lactamase (*blaNDM*), as well as colistin-resistance mechanisms involving mutations in the *pmrAB/pmrCAB* regulatory system, *eptA*-mediated lipid A modification, insertion sequence-mediated disruption of *lpxA*, *lpxC*, and *lpxD*, and other adaptive mutations (Beceiro et al., 2014; Moussa et al., 2023). The increasing prevalence of MDR and extensively drug-resistant strains contributes to substantial morbidity, mortality, prolonged hospitalization, and economic burden worldwide, underscoring the need for integrated genomic surveillance, strict infection control, antimicrobial stewardship, and development of novel therapeutic strategies, including new *β*-lactam/β-lactamase inhibitor combinations (Castillo-Ramírez, 2022; Lepak et al., 2023).

Beyond healthcare settings, ABC complex species have been detected in aquatic environments, livestock, wastewater treatment plants, and companion animals, indicating environmental and veterinary reservoirs that may facilitate zoonotic transmission and distribution of resistance determinants between non-clinical and clinical settings (Lysitsas et al., 2023; Benoit et al., 2024; Savin et al., 2024). Understanding their distribution across these reservoirs is critical for epidemiological surveillance and risk assessment (Benoit et al., 2024). Species-level identification enables tracking of dominant clones and resistance genes, strengthening outbreak investigation and containment strategies (Kang et al., 2023).

Although accurate identification is crucial for clinical management and infection control, conventional phenotypic methods frequently fail to reliably distinguish species within the ABC complex. This limitation necessitates the use of advanced molecular techniques to ensure precise classification and resistance profiling (Marinova-Bulgaranova et al., 2025). Comprehensive surveillance integrating clinical, environmental, and veterinary data is therefore essential to mitigate the global impact of the ABC complex (Benoit et al., 2024).

In this review, we will discuss innovative therapeutic strategies to combat MDR ABC complex infections. Prior to addressing treatment approaches, it is essential to examine the current methods for species identification and the underlying mechanisms of AMR within this complex. A comprehensive understanding of diagnostic strategies and resistance determinants is critical for informing targeted and effective therapeutic interventions, particularly in the context of emerging innovative treatment modalities.

## 2 Identification methods

Accurate identification of species within the ABC complex is essential for clinical diagnosis, epidemiology surveillance, and infection control. However, the high phenotypic and genotypic similarity among members of this complex poses significant challenges. This section reviews the available methods, from conventional techniques to advanced molecular and proteomic technologies, highlighting their principles, diagnostic performance, and limitations.

### 2.1 Preliminary identification methods with limited species-level resolution

Preliminary phenotypic methods are useful for initial screening but cannot reliably separate closely related ABC complex species due to overlapping metabolic traits.

Initial laboratory identification typically relies on colony morphology, growth characteristics and simple biochemical tests. Evaluation of colony color, size, and shape, along with growth on selective and differential media, assists in preliminary classification. *Acinetobacter* species characteristically grow on MacConkey agar as non-lactose-fermenting colonies, supporting early presumptive identification. Gram staining and microscopic examination provide essential preliminary information. Members of the genus are Gram-negative coccobacilli, and microscopic morphology combined with staining characteristics offers initial diagnostic guidance. Rapid biochemical assays further support identification: *Acinetobacter* species are typically oxidase-negative and catalase-positive, features that distinguish them from other Gram-negative rods (Püntener-Simmen et al., 2019; Abdel Hadi, 2025). Automated biochemical identification systems, including VITEK 2, Phoenix, and MicroScan, have streamlined identification by analyzing metabolic profiles. However, their accuracy for species-level identification within the ABC complex is limited. These systems often fail to reliably distinguish closely related species such as *A. baumannii*, *A. pittii*, and *A. nosocomialis* due to overlapping reactions (Wani et al., 2016; Marinova-Bulgaranova et al., 2025). Consequently, phenotypic methods are best suited for preliminary genus-level screening and must be confirmed by genotypic or proteomic approaches for definitive species identification within the ABC complex.

### 2.2 Targeted molecular methods with improved species discrimination

The limitations of conventional phenotyping have driven the development of a range of molecular techniques that offer higher resolution and specificity for species identification within the ABC complex.

#### 2.1.1 Single and multiplex PCR assays

Targeted PCR assays are widely used for rapid and specific identification. DNA gyrase subunit B (*gyrB)*, RNA polymerase beta-subunit (*rpoB)*, *blaOXA*-51-like, Recombinase A (*recA)* and 16S-23S rRNA have been proved valuable genes.

*gyrB* gene, which encodes the B subunit of DNA gyrase, has demonstrated high accuracy in differentiating common clinical species, including *A. baumannii*, *A. pittii*, and *A. nosocomialis*. A single-tube multiplex polymerase chain reaction (PCR) assay for *gyrB* offers a rapid, practical, and cost-effective alternative to conventional phenotypic identification (Albert et al., 2022). The RNA polymerase *β*-subunit (*rpoB*) is a robust phylogenetic marker. Short-signature *rpoB* sequencing enables rapid species identification suitable for routine diagnostics and links identification to rifampicin resistance profiling (La Scola et al., 2006; Marinova-Bulgaranova et al., 2025). Since *blaOXA-51-like* gene is intrinsic to *A. baumannii*, its detection by specific PCR serves as a reliable marker for confirming the identity of *A. baumannii* within the complex and is widely used in clinical and research settings (Woodford et al., 2006; Higgins et al., 2013). However, reliance on *blaOXA-51-like* detection alone may lead to misidentification, as exceptional cases have been reported. For example, Lee et al. (2012) described *blaOXA-51-like* genes in non-*baumannii* members of the ABC complex, while Zander et al. (2013) reported limitations related to atypical or variant *blaOXA-51-like* gene sequences. Therefore, *blaOXA-51*-based PCR should preferably be combined with additional targets such as *rpoB*, *gyrB*, *recA*, or 16S–23S rRNA intergenic spacer analysis for more reliable species-level identification (Lee et al., 2012; Zander et al., 2013). RecA is a multifunctional recombinase that orchestrates DNA damage repair and the SOS response in *Acinetobacter*, playing a central role in maintaining genomic stability. It directly impacts bacterial physiology by modulating biofilm formation, antibiotic resistance, and virulence. Its ability to activate error-prone DNA repair polymerases is a key driver of the emergence of antimicrobial resistance. Species-Specific PCR targeting *rpoB*, *recA*, and 16S-23S rRNA intergenic spacer assays also showed high sensitivity and specificity for distinguishing closely related species within clinical samples (Nowak and Kur, 1995; Chen et al., 2021).

### 2.3 Sequence- and genome-based methods for high-resolution differentiation

Genome-wide analyses such as ANI, dDDH, WGS, and cgMLST provide the highest species-level resolution by comparing large genomic regions, enabling accurate assignment even when single-gene markers are ambiguous.

#### 2.3.1 DNA–DNA hybridization (DDH)

Historically, DNA–DNA hybridization served as the gold standard for defining species boundaries within the ABC complex. DDH was instrumental in formally delineating *A. baumannii*, *A. calcoaceticus*, *A. pittii*, and *A. nosocomialis*, and contributed to the recognition of later species such as *A. seifertii* and *A. djikshoorniae* (Esen and Gözalan, 2020). Despite its historical importance, DDH is technically demanding, labor-intensive, poorly reproducible between laboratories, and has largely been superseded by whole-genome sequencing for taxonomic and diagnostic purposes (Rosselló-Mora, 2006; Albert et al., 2022).

#### 2.3.2 Sequence-based identification (16S rRNA, *rpoB*, and *gyrB*)

While the 16S rRNA gene is the most widely used phylogenetic marker for bacterial identification, its high sequence conservation within the ABC complex provides insufficient resolution to reliably discriminate between closely related species (Villalón et al., 2019). Due to limitations of 16S rRNA, sequencing protein-coding genes such as *rpoB* and *gyrB* is now recommended. These genes evolve faster and offer greater discriminatory power, allowing accurate species-level assignment. *gyrB* sequencing has been validated against whole-genome sequencing (WGS) and average nucleotide identity (ANI), confirming its reliability for identifying *A. baumannii* (Villalón et al., 2019; Albert et al., 2022; Nichols and Davenport, 2025).

#### 2.3.3 Average nucleotide identity (ANI)

ANI is a genome-wide measure of nucleotide similarity between two bacterial genomes and is now widely used for prokaryotic species identification. It provides a more reproducible and scalable alternative to conventional DNA–DNA hybridization, especially when whole-genome sequencing data are available (Konstantinidis and Tiedje, 2005; Richter and Rosselló-Móra, 2009). ANI is particularly valuable for the ABC complex because closely related species, including *A. baumannii*, *A. pittii*, *A. nosocomialis*, *A. seifertii*, and *A. dijkshoorniae*, may be difficult to distinguish using phenotypic tests or conserved single-gene markers. An ANI threshold of approximately 95–96% is generally accepted for species-level delineation, with values above this range supporting assignment to the same species (Richter and Rosselló-Móra, 2009; Kim et al., 2014; Ciufo et al., 2018). Therefore, ANI provides a robust and practical framework for confirming species identity and resolving taxonomic uncertainty within the ABC complex.

#### 2.3.4 Digital DNA–DNA hybridization (dDDH)

dDDH is an In silico method that estimates genome relatedness in a way that reflects traditional laboratory-based DNA–DNA hybridization. It preserves the taxonomic basis of conventional DDH while avoiding many of its limitations, including technical complexity, poor inter-laboratory reproducibility, and labor-intensive procedures (Meier-Kolthoff and Göker, 2019; Auch et al., 2010). A dDDH value of approximately 70% remains the commonly accepted threshold for species demarcation, with values at or above this level indicating that two isolates likely belong to the same species (Meier-Kolthoff and Göker, 2019; Auch et al., 2010). Together, ANI and dDDH provide complementary genome-based evidence for accurate species identification and taxonomic assignment among closely related *Acinetobacter* species (Richter and Rosselló-Móra, 2009; Meier-Kolthoff and Göker, 2019).

#### 2.3.5 Multilocus sequence typing (MLST) and core genome MLST (cgMLST)

MLST is a cornerstone technique for global epidemiological surveillance, particularly for tracking the spread of high-risk clones. Two main schemes exist for *A. baumannii*: the Oxford scheme (seven housekeeping genes) and the Pasteur scheme (which partially overlaps but provides more stable clonal assignments). MLST has identified globally disseminated, multidrug-resistant lineages (e.g., clonal complex 2, CC2; ST2) and revealed novel sequence types in both clinical and environmental reservoirs (Gaiarsa et al., 2019; Wareth et al., 2021; Choudhary and Shariff, 2025).

cgMLST enhances the standardization and inter-laboratory comparability of WGS data. By analyzing a defined core genome (e.g., 2,390 genes in *A. baumannii*), cgMLST provides higher discriminatory capacity than conventional MLST and PFGE, allowing the detection of microevolutionary changes and the separation of distinct transmission chains during hospital outbreaks (Higgins et al., 2017; Venditti et al., 2018; Li et al., 2022).

#### 2.3.6 Whole-genomic sequencing (WGS)

WGS now represents the highest-resolution approach for strain typing, outbreak investigation, and resistance prediction. It provides superior discriminatory power compared to PFGE and MLST, enabling the reconstruction of transmission pathways in healthcare settings and the identification of high-risk clones carrying carbapenemase genes (Woodford et al., 2006; Halachev et al., 2014). Furthermore, WGS can accurately predict antimicrobial susceptibility phenotypes and detect plasmid-mediated resistance determinants, supporting clinical management and surveillance (Fida et al., 2022).

### 2.4 Rapid and emerging diagnostic methods for clinical application

Recent technological advances offer faster and more decentralized diagnostic approaches, including CRISPR-Cas-based platforms, microarray-based assays, Fluorescence *In Situ* Hybridization (FISH), and cell wall binding (CBD)-based magnetic capture assays.

#### 2.4.1 Loop-mediated isothermal amplification (LAMP)

LAMP is a rapid, sensitive, and cost-effective isothermal amplification technique suitable for resource-limited settings. LAMP assays have been developed for direct detection of ABC complex species and associated carbapenem resistance genes (e.g., *blaOXA-23*). They demonstrate high analytic sensitivity (detection limits as low as 10 pg./μl DNA or 10^4^ CFU/mL), providing results within hours and offering a practical alternative to PCR-based methods, especially for point-of-care testing and environmental surveillance (Nayak et al., 2023; Sharma et al., 2024).

#### 2.4.2 High-resolution melting (HRM) analysis

HRM is a post PCR method that analyzes the melting behavior of double-stranded DNA. It enables rapid species discrimination by generating characteristics melting curve profiles without the need for sequencing or gel electrophoresis. HRM assays have been effectively integrated with multiplex PCR for simultaneous detection of resistance determinants (e.g., *blaOXA-23*, *AdeB*) in MDR *A. baumannii* and have shown high concordance with Pulsed-Field Gel Electrophoresis (PFGE) for outbreak monitoring (Woksepp et al., 2014; Wong et al., 2014; Sun et al., 2019).

#### 2.4.3 Metagenomic next-generation sequencing (mNGS)

mNGS enables culture-independent identification and resistance profiling directly from clinical specimens. By sequencing all microbial DNA in a sample and comparing reads to curated databases, mNGS accurately identifies species within the ABC complex without prior culture. When combined with machine learning models, mNGS can rapidly predict antimicrobial susceptibility, substantially reducing turnaround time compared to conventional Antimicrobial Susceptibility Testing (AST) (Ugolotti et al., 2016; Hu et al., 2023; Prakika et al., 2025).

#### 2.4.4 Proteomic identification (MALDI-TOF MS)

Matrix-assisted laser desorption ionization–time of flight mass spectrometry (MALDI-TOF MS) has revolutionized routine microbial identification in clinical laboratories. It analyzes the unique protein mass fingerprint of a bacterial isolate and compares it against a reference database. MALDI-TOF MS is exceptionally rapid (results in minutes), has a low per-sample cost after initial investment, and is now widely adopted for routine identification of *Acinetobacter* species. Studies report high accuracy for identifying *A. baumannii*, *A. pittii*, and *A. nosocomialis* using systems like the Bruker Biotyper and VITEK MS. For example, the Bruker Biotyper correctly identified 98.6% of *A. baumannii* isolates (Hsueh et al., 2014). However, the accuracy of MALDI-TOF MS is entirely dependent on the quality and comprehensiveness of the reference database. Without high-quality spectra from all relevant species (including newer ones like *A. seifertii* and *A. djikshoorniae*), the system may fail to identify or, worse, misidentify an isolate, potentially leading to incorrect clinical decisions. Continuous database updates are essential (Marí-Almirall et al., 2017; Bagudo et al., 2020).

#### 2.4.5 CRISPR-Cas-based platforms

CRISPR-Cas systems, particularly CRISPR-Cas12a, coupled with isothermal amplification [e.g., Recombinase Polymerase Amplification (RPA)], enable highly specific and sensitive detection of target genes. Multiplex RPA-CRISPR-Cas12a assays can detect both the intrinsic *blaOXA-51-like* (for *A. baumannii* identification) and acquired *blaOXA-23* (for carbapenem resistance) genes in under 90 min. These platforms show great promise as point-of-care tests (Zhou et al., 2025).

#### 2.4.6 Microarray-based assays

DNA microarrays allow simultaneous detection of multiple species and resistance determinants.

A microsphere-based array can differentiate up to 13 species within the ABC complex in <8.5 h, and targeted arrays can profile up to 91 resistance determinants in *A. baumannii* within 4 h (Palethorpe et al., 2026).

#### 2.4.7 Fluorescence *in situ* hybridization (FISH)

FISH uses fluorescently labeled DNA probes targeting species-specific regions (e.g., 16S rRNA) to visualize bacteria directly in clinical specimens. Genus-specific probes (e.g., Aci, ACA) allow rapid, highly specific detection directly from blood cultures, providing a result in hours without the need for culture (Asaadi et al., 2018; Barbosa et al., 2023).

#### 2.4.8 Cell wall-binding domain (CBD)-based magnetic capture assay

A novel diagnostic approach uses the cell wall-binding domain (CBD) of an *A. baumannii* phage endolysin conjugated to epoxy magnetic beads (AbCD–eMB) to selectively capture *A. baumannii* from samples. The method distinguishes *A. baumannii* from closely related ABC species by exploiting the specific interaction between AbCD and a Lysin motif (LysM)-containing peptidoglycan receptor on the *A. baumannii* cell wall. The assay provides rapid, culture-independent detection within 1 h with a detection limit of ~3.4 × 10^3^ CFU/mL and demonstrated effective capture with recovery rates of 72.5% in buffer and 55.7% in clinical sputum specimens. Authentication was confirmed by detecting the intrinsic *bla*OXA-51-like gene in all captured isolates. Overall, the platform offers a fast, highly specific, and sensitive method for discriminating *A. baumannii* from closely related ABC complex species, suitable for point-of-care diagnostics (Kim et al., 2026) (Table 1).

**Table 1:** Comparative value of identification methods for distinguishing ABC complex species.

Method	Specimen or sample type	Identified species	Main diagnostic use	Ability of discrimination	Sensitivity/specificity/accuracy	References
Conventional phenotypic tests	Pure culture/clinical isolate	*Acinetobacter* spp.	Preliminary genus-level identification	Low; cannot reliably separate *A. baumannii*, *A. pittii*, and *A. nosocomialis*	Useful for presumptive identification only	Püntener-Simmen et al. (2019) and Abdel Hadi (2025)
Automated biochemical systems: VITEK 2, Phoenix, MicroScan	Pure culture/clinical isolate	ABC complex members	Routine laboratory identification	Low to moderate; overlapping biochemical profiles limit species-level accuracy	Requires confirmation by molecular/proteomic methods	Wani et al. (2016) and Marinova-Bulgaranova et al. (2025)
gyrB PCR/sequencing	Clinical isolates	*A. baumannii*, *A. pittii*, *A. nosocomialis*	Rapid molecular identification	High; *gyrB* provides better separation than conserved markers	High accuracy for differentiating common clinical species	Albert et al. (2022)
rpoB sequencing	Clinical isolates	ABC complex species	Species-level sequencing	High; useful phylogenetic marker for closely related species	Suitable for routine diagnostics	La Scola et al. (2006) and Marinova-Bulgaranova et al. (2025)
blaOXA-51-like PCR	Clinical isolates	Mainly *A. baumannii*	Confirmation marker for *A. baumannii*	Moderate; useful but should not be used alone because exceptions exist	Reliable when combined with other targets	Woodford et al. (2006), Lee et al. (2012), and Higgins et al. (2013)
recA and 16S–23S rRNA intergenic spacer PCR	Clinical isolates	Closely related ABC species	Species-specific PCR identification	Moderate to high; improves separation of closely related species	Reported high sensitivity and specificity	Nowak and Kur (1995) and Chen et al. (2021)
16S rRNA sequencing	Clinical/environmental isolates	Broad bacterial identification	General bacterial identification	Low within ABC complex; too conserved for reliable species separation	Useful mainly for genus-level identification	Villalón et al. (2019)
rpoB and gyrB sequencing	Clinical isolates	*A. baumannii* and related ABC species	Sequence-based identification	High; protein-coding genes provide stronger resolution than 16S rRNA	*gyrB* validated against WGS and ANI	Villalón et al. (2019), Albert et al. (2022), and Nichols and Davenport (2025)
DDH	Reference strains/genomic DNA	ABC complex species	Historical species delineation	High historically, but not practical for routine identification	Former gold standard; labor-intensive and poorly reproducible	Richter and Rosselló-Móra (2009), Esen and Gözalan (2020), and Albert et al. (2022)
ANI	Whole-genome sequences	Closely related *Acinetobacter* species	Genome-based species delineation	Very high; useful for resolving taxonomic uncertainty	Species boundary around 95–96% ANI	Konstantinidis and Tiedje (2005), Richter and Rosselló-Móra (2009), Kim et al. (2014), and Ciufo et al. (2018)
dDDH	Whole-genome sequences	Closely related *Acinetobacter* species	Genome-based species confirmation	Very high	Species boundary around 70% dDDH	Meier-Kolthoff and Göker (2019) and Auch et al. (2010)
LAMP	Clinical/environmental samples or DNA extracts	ABC species and resistance genes such as *bla*OXA-23	Rapid point-of-care detection	Moderate; depends on target design	Detection limits as low as 10 pg./μL DNA or 10^4^ CFU/mL	Nayak et al. (2023) and Sharma et al. (2024)
HRM analysis	Clinical isolates/DNA extracts	MDR *A. baumannii* and resistance markers	Rapid species/resistance discrimination	Moderate to high; based on melting curve differences	High concordance with PFGE for outbreak monitoring	Woksepp et al. (2014), Wong et al. (2014), and Sun et al. (2019)
MLST	Clinical/environmental isolates	Sequence types and clonal complexes	Global epidemiological surveillance	Moderate; useful for lineage tracking but limited for fine outbreak resolution	Identifies high-risk clones such as CC2/ST2	Gaiarsa et al. (2019), Wareth et al. (2021), and Choudhary and Shariff (2025)
cgMLST	Whole-genome sequences	Closely related outbreak isolates	High-resolution outbreak typing	Very high; separates transmission chains and microevolutionary variants	Higher resolution than MLST and PFGE	Higgins et al. (2017), Venditti et al. (2018), and Li et al. (2022)
WGS	Clinical isolates/genomic DNA	ABC species, clones, resistance genes	Species ID, outbreak tracing, resistance prediction	Very high; strongest overall resolution	Superior to PFGE and MLST for transmission analysis	Woodford et al. (2006), Halachev et al. (2014), and Fida et al. (2022)
mNGS	Direct clinical specimens	ABC species and resistance profiles	Culture-independent diagnosis	High; depends on database quality and sequencing depth	Supports species detection and AST prediction	Ugolotti et al. (2016), Hu et al. (2023), and Prakika et al. (2025)
MALDI-TOF MS	Pure culture/clinical isolate	*A. baumannii*, *A. pittii*, *A. nosocomialis*	Rapid proteomic identification	Moderate to high; database-dependent	Bruker Biotyper identified 98.6% of *A. baumannii* isolates	Hsueh et al. (2014), Marí-Almirall et al. (2017), and Bagudo et al. (2020)
CRISPR-Cas12a with RPA	DNA extracts/clinical samples	*A. baumannii* and carbapenem resistance genes	Rapid molecular detection	High when species-specific targets are selected	Detects *bla*OXA-51-like and *bla*OXA-23 within 90 min	Zhou et al. (2025)
DNA microarray	Clinical isolates/DNA extracts	Multiple ABC species and resistance determinants	Multiplex species and resistance detection	High; detects multiple targets simultaneously	Differentiates up to 13 ABC species in <8.5 h; detects up to 91 resistance determinants in 4 h	Palethorpe et al. (2026)
FISH	Blood cultures/clinical specimens	*Acinetobacter* spp.	Direct visualization and rapid detection	Moderate; depends on probe specificity	Provides results within hours without culture	Asaadi et al. (2018) and Barbosa et al. (2023)
CBD-based magnetic capture assay (AbCD–eMB)	Buffer and clinical sputum specimens	*A. baumannii*	Rapid selective bacterial capture	High; separates *A. baumannii* using specific AbCD binding to a LysM-containing peptidoglycan receptor	Detection limit ~3.4 × 10^3^ CFU/mL; recovery 72.5% in buffer and 55.7% in sputum within 1 h	Kim et al. (2026)

## 3 Resistant mechanism

The ABC complex exhibits a remarkable ability to develop resistance to multiple classes of antimicrobial agents, which has significantly limited available treatment options in clinical settings. This resistance is mediated through several coordinated mechanisms, including enzymatic degradation of antibiotics, reduced membrane permeability, active efflux systems, and mutations in antibiotic target sites. The coexistence of multiple resistance mechanisms within a single strain contributes to the widespread emergence of MDR, extensively drug-resistant (XDR), and pan-drug-resistant (PDR) *Acinetobacter* strains worldwide (Mukhopadhyay et al., 2025; Pearl and Anbarasu, 2025).

### 3.1 Efflux pump mediated resistance

#### 3.1.1 Resistance-nodulation-cell division (RND) family

##### 3.1.1.1 AdeABC

**Figure 1:**
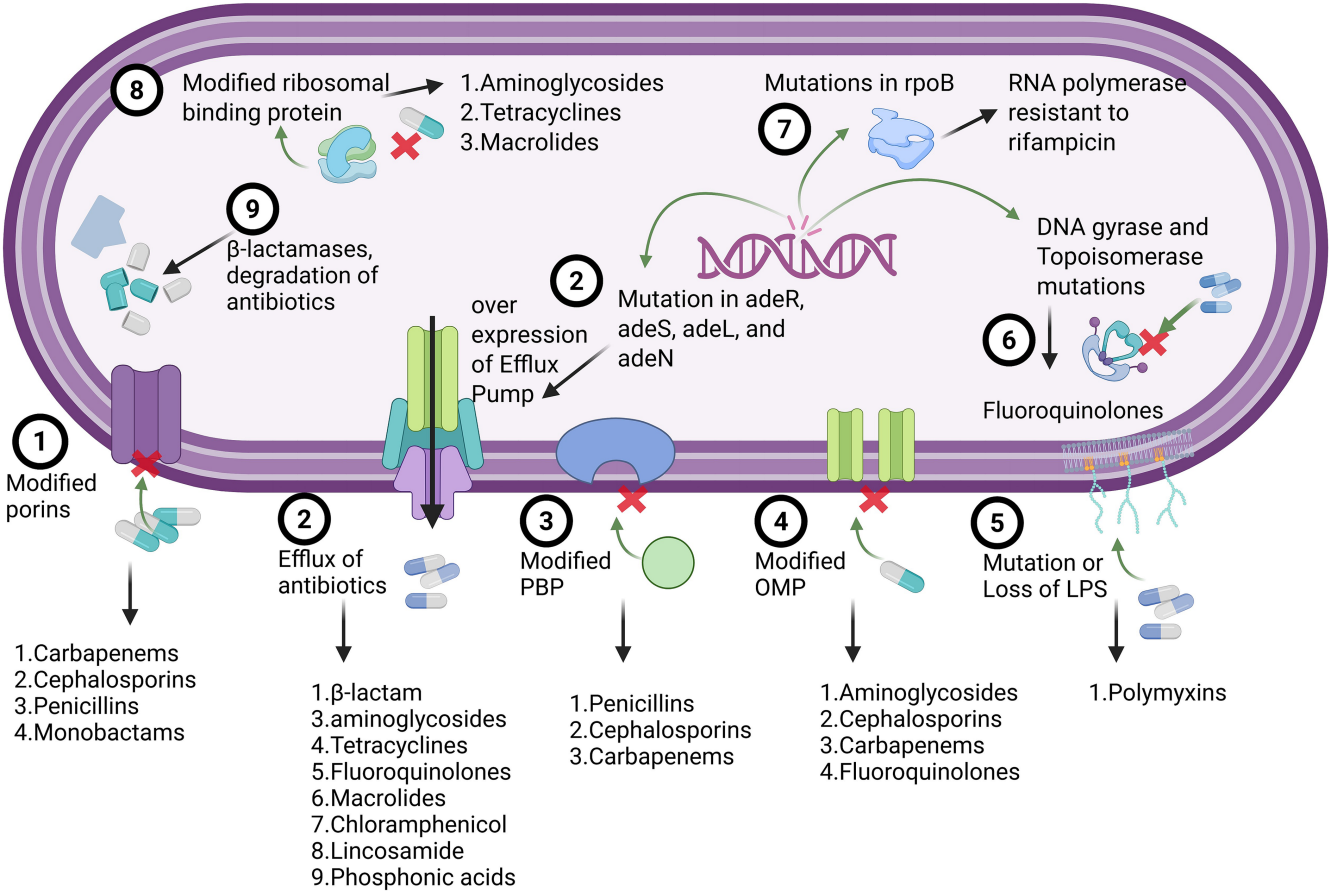
Schematic illustration of the major antibiotic resistance mechanisms in the ABC complex, including β-lactamase production, efflux pump activity, porin loss, gene mutation, ribosomal binding protein modification, target site modification, and membrane alterations.

The AdeABC efflux pump is one of the most clinically significant RND-type systems in *A. baumannii*, contributing predominantly to acquired multidrug resistance when overexpressed. It exhibits a broad substrate profile, including *β*-lactams, fluoroquinolones, tetracyclines, and aminoglycosides, and functions by actively extruding antibiotics from the bacterial cytoplasm, thereby lowering intracellular drug concentrations (Yoon et al., 2015; Lari et al., 2018; Tuo et al., 2023). The operon is tightly regulated by the *AdeRS* two-component regulatory system, and mutations in *adeR* or *adeS* (Figure 1) frequently result in constitutive overexpression in clinical isolates, particularly those exhibiting carbapenem resistance (Lari et al., 2018; Roy et al., 2021).

##### 3.1.1.2 AdeIJK

AdeIJK represents the intrinsic and evolutionarily conserved RND efflux system across the *Acinetobacter* genus (Darby et al., 2022, 2023). It is constitutively expressed and contributes to basal resistance against multiple antimicrobial classes, including β-lactams, chloramphenicol, tetracycline, and erythromycin (Damier-Piolle et al., 2008). Structurally, AdeIJK is composed of the inner membrane transporter AdeJ, the membrane fusion protein AdeI, and the outer membrane factor AdeK, forming a tripartite complex that spans the cell envelope. Its expression is negatively regulated by the TetR-type repressor *AdeN*; mutations in *adeN* lead to pump overexpression and enhanced (Damier-Piolle et al., 2008; Rosenfeld et al., 2012). However, excessive overexpression imposes a fitness cost and may be deleterious to the host bacterium, limiting its role in high-level acquired resistance while maintaining a critical contribution to intrinsic resistance and membrane homeostasis. Experimental inactivation of AdeIJK significantly increases antibiotic susceptibility, underscoring its essential role in maintaining the intrinsic resistome (Coyne et al., 2011; Leus et al., 2018).

##### 3.1.1.3 AdeFGH

The AdeFGH system constitutes another RND-type multidrug efflux pump, although its contribution is generally less pronounced compared with AdeABC and AdeIJK (Leus et al., 2020). The adeFGH operon encodes a tripartite complex comprising an RND transporter, a membrane fusion protein, and an outer membrane factor, facilitating substrate extrusion across both membranes (Coyne et al., 2010). Overexpression is commonly associated with mutations in *adeL*, a *LysR*-type transcriptional regulator located upstream of the operon, leading to increased transcription and enhanced resistance phenotypes. AdeFGH exhibits broad substrate specificity, including fluoroquinolones, tetracyclines, chloramphenicol, clindamycin, and certain dyes, and spontaneous mutants overexpressing the pump demonstrate elevated resistance to multiple antibiotics and biocides. Compensatory upregulation of AdeFGH has been observed following disruption of other efflux systems such as AdeABC, reflecting functional redundancy and regulatory cross-talk within the efflux network. Although widely distributed among clinical isolates, its expression is strain-dependent and often requires specific genetic or environmental triggers (Coyne et al., 2010; Leus et al., 2018).

Collectively, the RND efflux systems AdeABC, AdeIJK, and AdeFGH constitute a coordinated and hierarchically regulated network that underpins both intrinsic and acquired multidrug resistance in *A. baumannii*. Their activity not only reduces intracellular antibiotic accumulation but also influences membrane composition, stress responses, and biofilm formation, thereby exacerbating therapeutic challenges (Yoon et al., 2015).

#### 3.1.2 Major facilitator superfamily (MFS) efflux pumps

##### 3.1.2.1 AbaF

AbaF is a major facilitator superfamily (MFS) transporter specifically associated with Fosfomycin resistance in *A. baumannii*. It mediates resistance by actively exporting Fosfomycin from the bacterial cytoplasm, thereby reducing intracellular drug accumulation and limiting its inhibitory effect on peptidoglycan biosynthesis. Disruption of the *abaF* gene significantly increases susceptibility to Fosfomycin and is accompanied by reduced biofilm formation and attenuated virulence, indicating that *AbaF* contributes not only to AMR but also to pathogenic fitness. Moreover, exposure to fosfomycin increases *abaF* expression, and this increased expression is also observed in fosfomycin-resistant mutants, suggesting that *abaF* plays an adaptive role in bacterial survival under antibiotic stress (Sharma et al., 2017).

##### 3.1.2.2 AmvA

AmvA is another MFS efflux pump implicated in multidrug and biocide resistance. Elevated expression of amvA has been reported in environmental isolates resistant to disinfectants such as NANOSIL D2, a hydrogen peroxide–based formulation, suggesting its involvement in tolerance to oxidative biocides. Although its substrate range is less clearly defined compared with RND pumps, AmvA, like other MFS transporters, contributes to multidrug resistance by exporting structurally diverse antimicrobial agents and thereby lowering intracellular drug concentrations (Kumar et al., 2020; Rostami et al., 2021; Lekshmi et al., 2024).

##### 3.1.2.3 CraA

CraA (Chloramphenicol-Resistance *A. baumannii*) is a poly-specific MFS efflux pump that plays a significant role in multidrug resistance. Structurally related to the MdfA transporter of *Escherichia coli*, CraA contains distinct amino acid residues that determine substrate specificity. It functions as a proton motive force–dependent transporter and possesses a hydrophobic cavity accessible from the cytoplasm, which facilitates substrate binding and extrusion. Critical residues such as E338 and Y42 have been identified as key determinants of substrate recognition; E338 is essential for norfloxacin transport, whereas Y42 is crucial for phenicol binding (Foong et al., 2025).

##### 3.1.2.4 Tet family (TetB efflux pump)

Members of the Tet (Ten-Eleven Translocation) family of MFS transporters are primarily associated with tetracycline resistance. These pumps actively expel tetracycline from the bacterial cell, preventing the drug from reaching ribosomal target sites (Lekshmi et al., 2024). Among them, TetB is the predominant determinant of tetracycline resistance in *A. baumannii*. The presence of the tet(B) gene strongly correlates with elevated minimum inhibitory concentrations (MICs) for tetracycline and minocycline, demonstrating its functional importance (Rosenfeld et al., 2012; Lomovskaya et al., 2018).

#### 3.1.3 Other efflux systems

AbeM, a member of the multidrug and toxic compound extrusion (MATE) family, has been implicated in resistance of *Acinetobacter baumannii* to disinfectants, including hydrogen peroxide–based formulations. Upregulation of the *abeM* gene has been observed in isolates resistant to disinfectants such as NANOSIL D2 and OPIDEX OPA, supporting its role in biocide tolerance (Rostami et al., 2021). Furthermore, exposure of clinical isolates to subinhibitory concentrations of disinfectants has been associated with increased expression of *abeM* and other efflux pump genes, indicating that environmental and hospital disinfectant pressure can induce efflux-mediated adaptive responses (Lin et al., 2017). Beyond its role in disinfectant tolerance, AbeM contributes to broad-spectrum antibiotic resistance in *A. baumannii*. As a MATE transporter, it utilizes ion gradients to actively export structurally diverse antimicrobial agents, thereby reducing intracellular drug accumulation. This activity supports the MDR phenotype frequently observed in clinical settings (Sharma et al., 2017; Zack et al., 2024).

##### 3.1.3.1 Small multidrug resistance (SMR) efflux pump AbeS

The SMR family includes compact membrane transporters that export toxic compounds from bacterial cells and contribute to antimicrobial and biocide tolerance (Srinivasan et al., 2009; Kornelsen and Kumar, 2021). In *A. baumannii*, AbeS is a chromosomally encoded SMR efflux pump that contributes to low-level resistance against multiple structurally diverse compounds. Functional characterization showed that AbeS can export antimicrobial agents and toxic compounds including chloramphenicol, erythromycin, ciprofloxacin, novobiocin, dyes, detergents, and antiseptics (Srinivasan et al., 2009). Structural and mechanistic studies further support that AbeS has broad substrate recognition capacity, which explains its contribution to multidrug and biocide resistance (Lytvynenko et al., 2016). Although AbeS is generally less dominant than major RND efflux systems such as AdeABC, it may still support survival under antibiotic and disinfectant pressure when combined with *β*-lactamases, membrane permeability changes, biofilm formation, and other efflux systems (Kornelsen and Kumar, 2021; Kyriakidis et al., 2021).

### 3.2 *β*-Lactamase–mediated resistance

#### 3.2.1 Class A beta-lactamses (*β*-lactamases)

Class A *β*-lactamases include extended-spectrum *β*-lactamases (ESBLs), non-carbapenemase *β*-lactamases, and less frequently reported Class A carbapenemases. ESBLs contribute to resistance against third-generation cephalosporins and aztreonam. Among these, *blaTEM, blaCTX-M* (Cefotaxim-Munich), and *blaSHV* (Sulfhydryl Variable) are widely reported in Gram-negative bacteria and have also been detected in *A. baumannii* (Chaudhary et al., 2023; Elsayed et al., 2024). In the ABC complex, *blaTEM* is often reported as the predominant ESBL gene, followed by *bla*SHV and *bla*CTX-M, although prevalence varies geographically (Smiline et al., 2018). Other Class A ESBL genes, including *bla*PER (Pseudomonas extended resistance) and *bla*VEB (Vietnamese extended-spectrum *β*-lactamase), also contribute to β-lactam resistance in *A. baumannii*. The *bla*PER gene has been reported in extensively drug-resistant isolates in North India (15.3%) and at higher frequencies among MDR isolates in Tehran (61.5%), whereas it was absent in some regions such as Kerman, Iran (Hassan et al., 2020; Nageeb et al., 2023). Similarly, *bla*VEB shows geographic heterogeneity, with detection reported in Tehran (35.3%) and Kerman (13.7%) but absence in some Egyptian clinical isolates (Hassan et al., 2020; Nageeb et al., 2023; Höfken et al., 2025). In addition to ESBLs, Class A carbapenemases, such as *bla*KPC (*Klebsiella pneumoniae* carbapenemase) and *bla*GES (Guiana extended-spectrum), have occasionally been reported in *Acinetobacter* spp. and may contribute to carbapenem resistance, although they are less common than OXA-type Class D carbapenemases and metallo-*β*-lactamases in the ABC complex (Rahi, 2024; Sathyanesan et al., 2024).

#### 3.2.2 Class B metallo-*β*-lactamases

Metallo-*β*-lactamases (MBLs) confer high-level resistance to carbapenems and are zinc-dependent enzymes capable of hydrolyzing nearly all β-lactams except monobactams. The blaNDM gene has been identified in multiple Acinetobacter species, including *A. baumannii*, *A. nosocomialis*, and *A. pittii*, particularly in hospital settings in South America and India (Brasiliense et al., 2019; Sathyanesan et al., 2024). blaNDM is frequently located on plasmids associated with the composite transposon Tn125, facilitating interspecies dissemination. Similarly, blaVIM (Verona integron-encoded metallo-β-lactamase) is commonly embedded within class 1 integrons, enhancing its capture and expression (Nikibakhsh et al., 2021). blaIMP (imipenemase) represents another clinically significant MBL found in the ABC complex and is also integron-associated, contributing to regional and global spread (Sathyanesan et al., 2024). blaVIM and blaNDM occur with region-dependent variability (Mabrouk et al., 2020; Rahi, 2024).

#### 3.2.3 Class C *β*-lactamases (*blaADC*)

The *blaADC* genes encode Acinetobacter-derived cephalosporinases (Class C *β*-lactamases) and are intrinsic to the ABC complex. These enzymes confer resistance to broad-spectrum cephalosporins and form part of a large and diverse *β*-lactamase repertoire within *Acinetobacter* spp (Périchon et al., 2014; Shin and Park, 2017). The expression of *blaADC* can be significantly enhanced by the insertion sequence ISAba1 located upstream of the gene, which provides a strong promoter and leads to elevated cephalosporin resistance. Epidemiologically, *blaADC* is highly prevalent among *A. baumannii* isolates and is frequently associated with MDR phenotypes.

#### 3.2.4 Class D *β*-lactamases (oxacillinases, OXA-Type *β*-lactamases)

Class D *β*-lactamases (oxacillinases, OXA-type) are the most important carbapenemases in the ABC complex. These enzymes hydrolyze carbapenems with variable efficiency depending on the specific OXA subfamily (Evans and Amyes, 2014; Yoon and Jeong, 2021). Several *blaOXA* genes are intrinsic or naturally occurring in *A. baumannii*, particularly blaOXA-51-like variants, whereas others, such as *blaOXA-23* and *blaOXA-24/40* are often acquired and strongly associated with high-level carbapenem resistance (Périchon et al., 2014). OXA enzymes are typically lipidated and associated with the outer membrane, facilitating their incorporation into outer membrane vesicles (OMVs). This vesicle-mediated dissemination enhances horizontal spread of resistance determinants within hospital environments (Capodimonte et al., 2025). Clinically, the presence of OXA-type carbapenemases severely limits therapeutic options and is strongly correlated with carbapenem-resistant *A. baumannii* (CRAB) infections (Tietgen et al., 2019; Ejaz et al., 2022). Recent studies have identified novel variants such as *OXA-679* and *OXA-822*, expanding the known diversity of OXA enzymes. Phylogenetic analyses indicate that *blaOXA* genes have ancient evolutionary origins, with ongoing diversification contributing to emerging resistance phenotypes (Tian et al., 2018; Tietgen et al., 2019).

### 3.3 Target site alterations

#### 3.3.1 Fluoroquinolone resistance

In *A. baumannii*, resistance to fluoroquinolones is primarily driven by mutations in the quinolone resistance-determining regions (QRDRs) of the *gyrA* and *parC* genes, encoding DNA gyrase and topoisomerase IV, respectively (Geisinger et al., 2019). These enzymes are the principal targets of fluoroquinolones. Point mutations in *gyrA* (commonly *Ser83* substitutions) and *parC* (commonly *Ser80* substitutions) induce structural alterations in the drug-binding pocket, reducing fluoroquinolone affinity and resulting in high-level resistance (Martínez-Trejo et al., 2022). The accumulation of mutations in both targets is associated with progressively elevated MICs. In many clinical isolates, target-site mutations coexist with additional mechanisms, including efflux pump overexpression and plasmid-mediated determinants such as aac(6′)-Ib-cr, further strengthening the resistance phenotype (Roy et al., 2021; Aboelenin et al., 2024).

#### 3.3.2 Sulbactam resistance (PBP3 alterations)

Unlike most *β*-lactamase inhibitors, sulbactam possesses intrinsic antibacterial activity against *Acinetobacter baumannii* due to its ability to directly bind penicillin-binding proteins (PBPs), particularly PBP1 and PBP3, thereby inhibiting peptidoglycan synthesis. This unique dual role as both a β-lactamase inhibitor and an active antibacterial agent has made sulbactam-based therapies important treatment options against MDR *A. baumannii* (Penwell et al., 2015).

Resistance or reduced susceptibility to sulbactam-based therapies, including sulbactam–durlobactam, has been associated with mutations in penicillin-binding protein 3 (PBP3), a key enzyme involved in peptidoglycan synthesis, the primary target of sulbactam in *A. baumannii* (Moussa et al., 2023). Alterations such as A515V and T526S within the *ftsI* gene encoding PBP3 can modify the antibiotic-binding region, reducing the affinity of sulbactam for its target while largely preserving protein structure (Naha et al., 2021). These target-site modifications may diminish the antibacterial activity of sulbactam even in the presence of durlobactam, which functions primarily as a *β*-lactamase inhibitor. In clinical isolates, PBP3 mutations may occur alongside additional resistance determinants, including carbapenemase production and efflux pump overexpression, reflecting the multifactorial nature of β-lactam resistance in *A. baumannii* (Nageeb et al., 2023; Höfken et al., 2025).

#### 3.3.3 Tigecycline resistance

Reduced susceptibility to tigecycline has been linked to mutations in regulatory and transcription-associated genes, including *adeS*, *rpoB*, and *rrf* (Hua et al., 2021). Mutations in *adeS*, part of the AdeRS two-component regulatory system, can lead to constitutive overexpression of the AdeABC efflux pump, lowering intracellular tigecycline concentrations. Alterations in *rpoB*, encoding the *β*-subunit of RNA polymerase (Figure 1), influence global transcriptional regulation and may modulate the expression of resistance-associated genes, including methyl-transferases. Similarly, mutations in *rrf*, which encodes the ribosome recycling factor, affect translational efficiency and ribosomal dynamics, contributing indirectly to reduced tigecycline susceptibility. These findings demonstrate that tigecycline resistance may arise not only from direct target modification but also from broader regulatory adaptations that reshape bacterial physiology (Moland et al., 2008; Yang et al., 2023).

#### 3.3.4 Chloramphenicol resistance (*CatB8*)

Chloramphenicol resistance in *A. baumannii* can be mediated by *CatB8*, a chloramphenicol acetyltransferase. CatB8 enzymatically acetylates chloramphenicol to produce an inactive derivative, thereby preventing effective binding to the 50S ribosomal subunit and restoring protein synthesis. Structural analyses reveal that CatB8 possesses multiple chloramphenicol-binding sites per monomer, enhancing its catalytic efficiency. Specific amino acid residues within the active site are critical for substrate recognition and acetyl group transfer, underscoring the structural basis of this resistance mechanism (Liao et al., 2024).

### 3.4 Horizontal gene transfer and mobile genetic elements

#### 3.4.1 Horizontal gene transfer (HGT)

Horizontal gene transfer (HGT) is a major driver of antimicrobial resistance dissemination in the ABC complex because it enables *Acinetobacter* spp. to acquire resistance genes from other strains or bacterial species through mobile genetic elements such as plasmids, transposons, integrons, and genomic islands (Noel et al., 2022; Jeon et al., 2023). Unlike resistance caused only by point mutations, HGT allows bacteria to obtain complete resistance determinants, including carbapenemase genes, aminoglycoside-modifying enzyme genes, and other antimicrobial resistance genes that can spread rapidly in hospital environments. This process contributes to the emergence and persistence of multidrug-resistant *A. baumannii* lineages, particularly when resistance genes are carried on transferable plasmids or embedded within larger resistance islands (Noel et al., 2022; Sánchez-Urtaza et al., 2024).

#### 3.4.2 Insertion sequences

Insertion sequences are small mobile DNA elements that contribute to resistance by mobilizing nearby genes and by altering the expression of resistance determinants. In *A. baumannii*, *ISAba1* is especially important because it can insert upstream of *β*-lactamase genes and provide strong promoter sequences that increase gene expression. This mechanism has been associated with enhanced expression of resistance genes such as *bla*OXA-23, *bla*OXA-51-like, and *bla*ADC, thereby contributing to clinically significant β-lactam and carbapenem resistance. Therefore, insertion sequences should be discussed separately from general HGT because they not only participate in gene mobility but can also directly increase resistance gene expression (Mugnier et al., 2009; Noel et al., 2022).

### 3.5 Outer membrane permeability alterations

#### 3.5.1 Outer membrane protein alterations (OmpA)

OmpA is a major outer membrane protein in *A. baumannii*and plays a central role in AMR, virulence, and biofilm. Structurally, OmpA consists of an N-terminal eight-stranded β-barrel domain inserted into the outer membrane and a C-terminal periplasmic domain that interacts non-covalently with peptidoglycan. The β-barrel contains four extracellular loops that directly interface with the external environment, while the C-terminal region contributes to membrane stability and permeability control. Polymorphisms are frequently concentrated within the extracellular loops and C-terminal regions, suggesting adaptive evolution under clinical selective pressures. Functionally, OmpA acts as a slow porin, modulating the influx of small hydrophilic molecules, including certain antibiotics, thereby contributing to the MDR phenotype (Viale and Evans, 2020). Disruption of OmpA has been associated with reduced MICsof several antibiotics, such as chloramphenicol, aztreonam, and nalidixic acid (Figure 1), supporting its contribution to resistance (Smani et al., 2014). Additionally, the OmpA-like domain has been implicated in interactions with RND efflux systems, suggesting coordinated regulation of permeability and efflux mechanisms (Kwon et al., 2017).

#### 3.5.2 Porin loss or modification

CarO (carbapenem-associated outer membrane protein) is another outer membrane porin associated particularly with carbapenem susceptibility in *A. baumannii*. It forms β-strand–rich channels that allow the entry of carbapenems, including imipenem and meropenem (Mussi et al., 2005; Siroy et al., 2005). Loss, mutation, or reduced expression of CarO decreases membrane permeability and limits antibiotic influx, thereby contributing to carbapenem resistance (Fonseca et al., 2013; Zhu et al., 2019). Insertional inactivation of the *CarO* gene by mobile genetic elements is a common mechanism observed in resistant clinical isolates (Mussi et al., 2005). Structurally, CarO forms monomeric channels with slight cation selectivity and is not exclusively specific to imipenem (Siroy et al., 2005). Two principal allelic variants, *CarOa* and *CarOb,* have been described; *CarOb* demonstrates greater specificity for imipenem, indicating that sequence variation can influence substrate affinity and transport efficiency (Catel-Ferreira et al., 2011). In addition to porin loss, modifications of lipid A and complete loss of lipopolysaccharide (LPS) (Figure 1) can further reduce outer membrane permeability. Such alterations can stabilize the membrane and impair the binding and penetration of antibiotics such as colistin, thereby contributing to resistance (Jiang et al., 2020) (Table 2).

**Table 2:** Summary of major antibiotic resistance mechanisms and associated genes in ABC complex.

Resistance mechanism	Gene	Breif description	References
Intrinsic class C *β*-lactamase	*blaADC*	Chromosomal cephalosporinase; overexpression (often via *ISAba1*) increases resistance to extended-spectrum cephalosporins.	Périchon et al. (2014)
Class D *β*-lactamases (OXA-type)	*blaOXA-51-like*	Intrinsic carbapenemase of *A. baumannii*; baseline carbapenem hydrolysis enhanced by upstream insertion sequences.	Evans and Amyes (2014)
	*blaOXA-23*	Acquired carbapenemase widely disseminated globally; major driver of CRAB infections.	Tietgen et al. (2019)
	*blaOXA-24/40*	Plasmid-associated carbapenemase contributing to high-level carbapenem resistance.	Evans and Amyes (2014)
	*blaOXA-58*	Carbapenem-hydrolyzing enzyme frequently plasmid-borne; associated with hospital outbreaks.	Poirel and Nordmann (2006)
	*blaOXA-143*	Emerging OXA variant reported mainly in South America; confers carbapenem resistance.	Higgins et al. (2013
	*blaOXA-235*	OXA-235 family enzyme with carbapenemase activity; increasingly reported globally.	Higgins et al. (2013
Metallo-β-lactamase (class B)	*blaNDM-1*	Zinc-dependent carbapenemase; plasmid-mediated and highly transmissible.	Bogaerts et al. (2013)
	*blaNDM-5*	Variant of NDM with enhanced carbapenem hydrolysis.	Iimura et al. (2020)
	*blaVIM*	Integron-associated MBL conferring broad β-lactam resistance.	Walsh (2005)
	*blaIMP*	One of the earliest MBLs; globally disseminated in MDR isolates.	Queenan and Bush (2007)
Class A carbapenemase	*blaKPC*	Serine carbapenemase occasionally reported in *A. baumannii*; confers high-level β-lactam resistance.	Queenan and Bush (2007)
ESBLs	*blaTEM*	Hydrolyzes penicillin’s and third-generation cephalosporins.	Smiline et al. (2018)
	*blaSHV*	ESBL contributing to cephalosporin resistance.	Smiline et al. (2018)
	*blaCTX-M*	Potent ESBL targeting cefotaxime; increasingly detected worldwide.	Smiline et al. (2018)
Aminoglycoside-modifying enzymes	*aph(3′)-VI*	Phosphorylates aminoglycosides, inactivating the drug.	Ramirez and Tolmasky (2010)
	*aac(6′)-Ib*	Acetylates aminoglycosides; variant aac(6′)-Ib-cr also affects fluoroquinolones.	Robicsek et al. (2006)
	*aadA*	Adenylates streptomycin and spectinomycin.	Ramirez and Tolmasky (2010)
16S rRNA methyltransferase	*armA*	Methylates 16S rRNA, causing high-level pan-aminoglycoside resistance.	Aboelenin et al. (2024)
Fluoroquinolone target modification	*gyrA*	QRDR mutation reduces fluoroquinolone binding to DNA gyrase.	Vila et al. (1997)
	*parC*	Mutation alters topoisomerase IV, increasing resistance level.	Vila et al. (1997)
Plasmid-mediated quinolone resistance	*qnrA*	Protects DNA gyrase from fluoroquinolone inhibition.	Robicsek et al. (2006)
	*qnrB*	Plasmid-mediated quinolone resistance determinant.	Robicsek et al. (2006)
	*qnrS*	Confers low-level quinolone resistance.	Robicsek et al. (2006)
Sulbactam resistance (target alteration)	*pbp3 (ftsI)*	Mutations reduce sulbactam binding affinity.	Naha et al. (2021)
Tigecycline resistance (regulatory)	*adeRS*	Mutations cause overexpression of AdeABC efflux pump.	Hua et al. (2021)
	*rpoB*	Alters transcriptional regulation affecting susceptibility.	Hua et al. (2021)
	*Rrf*	Affects ribosome recycling and tigecycline response.	Hua et al. (2021)
RND Efflux pump	*adeA*	Membrane fusion component of AdeABC.	Damier-Piolle et al. (2008)
	*adeB*	Multidrug transporter of AdeABC; primary efflux component.	Damier-Piolle et al. (2008)
	*adeC*	Outer membrane channel of AdeABC.	Damier-Piolle et al. (2008)
	*adeI*	Component of AdeIJK efflux system.	Damier-Piolle et al. (2008)
	*adeJ*	Transporter component of AdeIJK.	Damier-Piolle et al. (2008)
	*adeK*	Outer membrane protein of AdeIJK.	Damier-Piolle et al. (2008)
	*adeF*	Component of AdeFGH efflux system.	Coyne et al. (2010)
	*adeG*	Transporter component of AdeFGH.	Coyne et al. (2010)
	*adeH*	Outer membrane factor of AdeFGH.	Coyne et al. (2010)
MFS Efflux pump	*abaF*	Exports Fosfomycin; contributes to resistance and virulence.	Sharma et al. (2017)
	*amvA*	Associated with multidrug and disinfectant resistance.	Rostami et al. (2021)
	*tet(B)*	Tetracycline efflux pump; major determinant of tetracycline resistance.	Lomovskaya et al. (2018)
MATE Efflux pump	*abeM*	Ion-dependent multidrug efflux pump contributing to MDR phenotype.	Lin et al. (2017)
Outer membrane permeability	*carO*	Loss reduces carbapenem uptake.	Mussi et al. (2005)
	*ompA*	Modulates permeability and biofilm-associated resistance.	Smani et al. (2014)
Colistin resistance (lipid A modification/LPS loss)	*pmrA*	Regulator activating lipid A modification genes.	Adams et al. (2009)
	*pmrB*	Sensor kinase mediating colistin resistance signaling.	Adams et al. (2009)
	*lpxA*	Mutation leads to LPS loss and colistin resistance.	Adams et al. (2009)
	*lpxC*	Involved in lipid A biosynthesis; mutations confer colistin resistance.	Adams et al. (2009)
	*lpxD*	Mutation disrupts LPS synthesis, causing resistance.	Adams et al. (2009)
Chloramphenicol resistance	*catB8*	Acetylates chloramphenicol, preventing ribosomal binding.	Liao et al. (2024)

## 4 Treatment

Treatment of infections caused by the ABC complex is challenging because many clinical isolates are multidrug-resistant or extensively drug-resistant. Conventional antibiotic therapy remains important, but therapeutic failure is common when resistance to carbapenems, colistin, or other last-line agents is present. Therefore, alternative and adjunctive strategies are increasingly being explored. The following section discusses major emerging treatment approaches, including phage therapy, engineered endolysins, monoclonal antibodies, vaccine-based immunotherapy, and CRISPR-Cas-based antimicrobial strategies.

### 4.1 Phage therapy

#### 4.1.1 Mechanism and antibacterial activity

**Figure 2:**
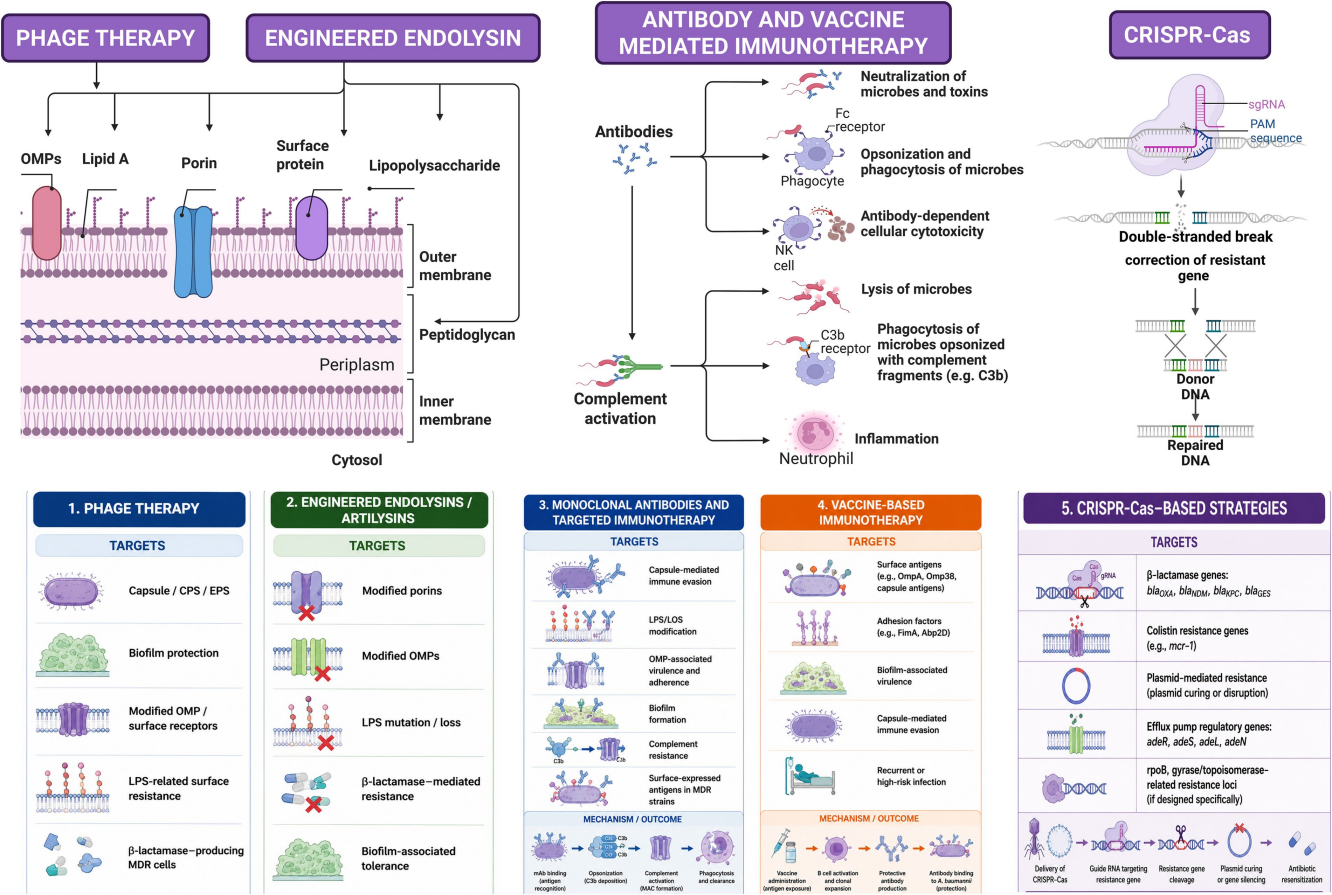
Emerging therapeutic strategies against the *Acinetobacter baumannii–calcoaceticus* (ABC) complex, including phages, engineered lysins, monoclonal antibodies, vaccines, and CRISPR-Cas approaches targeting resistance, biofilms, and virulence determinants.

Bacteriophage therapy has emerged as a promising alternative strategy for combating multidrug-resistant *Acinetobacter baumannii*, particularly in infections where conventional antibiotics demonstrate limited efficacy. Phages exhibit highly specific antibacterial activity by recognizing surface structures of bacterial cells and initiating a lytic replication cycle that ultimately results in bacterial destruction. In *A. baumannii*, effective phage infection typically begins with the recognition of capsular polysaccharides (CPS) (Figure 2), which serve as major virulence determinants and key mediators of immune evasion (Gordillo Altamirano et al., 2021; Chen et al., 2022a, 2022b).

Many bacteriophages encode tail-associated depolymerases that bind to and enzymatically degrade CPS, exposing the bacterial outer membrane and enabling irreversible adsorption, genome injection, and productive infection (Oliveira et al., 2017; Knirel et al., 2020). The extensive structural diversity of CPS among clinical isolates drives co-evolution of phage tail fibers, particularly those harboring pectate lyase domains responsible for capsule binding and degradation (Oliveira et al., 2017). A representative example is Acinetobacter phage Petty, which encodes a depolymerase capable of degrading host exopolysaccharides (EPS), as demonstrated by reduced viscosity and the release of reducing sugar ends consistent with hydrolase activity; this function enhances biofilm penetration and improves infection efficiency in encapsulated cells (Hernandez-Morales et al., 2018).

Biofilm disruption represents a significant therapeutic advantage because biofilms protect *A. baumannii* from antibiotics and immune responses; phage-mediated EPS degradation increases bacterial accessibility to lytic activity and antimicrobial agents, thereby improving treatment outcomes (Grygiel et al., 2024). Phage vB_AbaM_PhT2 has demonstrated protective effects in human cell line models by reducing infection-associated cellular damage, supporting its capacity to disrupt biofilms and mitigate host tissue injury (Styles et al., 2020).

Bacterial Lysis is mediated by phage-encoded holins and endolysins; notably, Acinetobacter phage Petty lacks canonical spanins yet disrupts the outer membrane through a lysis cassette encoding a class I holin and a single-subunit endolysin (Hernandez-Morales et al., 2018). Therapeutic efficacy is enhanced through synergistic interactions, including cooperation with the complement system to target both encapsulated and non-encapsulated phenotypes (Chen et al., 2022a, 2022b) and phage–antibiotic synergy, as demonstrated by vB_AbaS_SA1, which reduces the minimum effective antibiotic concentration required for bacterial killing in combination regimens (Rastegar et al., 2024).

#### 4.1.2 Therapeutic development and clinical challenges

Genomic analyses of phages targeting the ABC complex reveal narrow host ranges with high genomic synteny across geographically distinct isolates, reflecting conserved functional modules, particularly tail fiber genes containing pectate lyase domains essential for capsule specificity (Oliveira et al., 2017). *In vitro* and *in vivo* investigations confirm strong lytic activity and environmental stability; the JHA phage achieves up to 99% lysis of MDR strains within 18 h and remains stable across varied pH and temperature ranges, with optimal performance at pH 7 and 37 °C (Styles et al., 2020), while pIsf-AB02 rapidly reduces host cell density within 30 min and maintains stability under physiological conditions (Sisakhtpour et al., 2022). Phage-derived lysins such as Abp013 demonstrate potent activity against MDR strains, effective biofilm penetration, and maintained activity in human serum, indicating suitability for systemic therapeutic application (Chu et al., 2022).

To address host range limitations, rationally designed phage cocktails and computational pipelines integrating genomic screening and host-range prediction have been developed (Nawaz et al., 2024), alongside engineered variants such as Ab105-2phiΔCI404ad, which exhibits extended host range and enhanced antibiofilm efficacy (Blasco et al., 2022). Clinical translation has demonstrated feasibility, including successful personalized phage therapy for carbapenem-resistant *A. baumannii* lung infection (Tan et al., 2021), although regulatory standardization, manufacturing control, individualized matching, and the emergence of phage-resistant variants remain ongoing challenges.

Complementary to whole-phage therapy, engineered depolymerases and artilysins target CPS, LPS (Figure 2), and EPS to disrupt biofilms and restore immune susceptibility (Shahed-al-mahmud et al., 2021; Chen et al., 2022a, 2022b; Islam et al., 2022), while depolymerases such as Dpo71 enhance colistin activity by destabilizing the outer membrane and improving antibiotic binding, and DpoMK34 promotes complement-mediated serum killing, with efficacy demonstrated in zebrafish and *Galleria mellonella* infection models (Vukotic et al., 2020; Abdelkader et al., 2022).

### 4.2 Engineered endolysins

#### 4.2.1 Antibacterial mechanism

Engineered endolysins have gained increasing attention as promising therapeutic agents against *A. baumannii*. Through protein engineering approaches such as fusion with membrane-destabilizing peptides, domain shuffling, and chemical modification, these lysins can overcome the outer membrane barrier of Gram-negative bacteria. As a result, they can exert potent bactericidal activity against clinically relevant strains (Defraine et al., 2016; Schmelcher and Loessner, 2021; Lim et al., 2022). The DS-PA90 achieved complete eradication of *A. baumannii* at low concentrations and demonstrated superior activity compared with its parental enzyme PA90 (Lim et al., 2022). Additional engineered lysins, including LysJEP8 derived from *Escherichia* phage JEP8 and LysECD7 modified with lipopolysaccharide-interacting peptides, have shown significant antimicrobial activity against MDR isolates (Rastegar et al., 2024).

Structural optimization further improves stability and catalytic efficiency, as demonstrated by AbLys1 from phage AbTZA1, whose characterized catalytic and binding domains contribute to potent and selective lytic activity (Premetis et al., 2023), while chemical modifications such as fatty acid derivatization enhance pharmacokinetic properties and in vivo therapeutic efficacy of LysECD7 derivatives. Recombinant lysins with broadened antimicrobial spectra, including Ablysin and Gp105, degrade peptidoglycan structures in multiple MDR pathogens while maintaining favorable safety profiles (Kim et al., 2020; Li et al., 2024).

Engineered lysins exert antibacterial activity against *A. baumannii* by penetrating the outer membrane and enzymatically degrading the peptidoglycan layer (Figure 2) of the bacterial cell wall. Their activity can be improved by fusing them with membrane-disrupting peptides, such as the LysMK34–cecropin A construct, which enhances antibacterial activity even against colistin-resistant strains (Islam et al., 2022). LysAB2-KWK demonstrates a 100,000-fold increase in activity against *A. baumannii*, underscoring the importance of C-terminal membrane-permeabilizing domains in antimicrobial potency (Khan et al., 2021), while artilysin Art-175 effectively eliminates actively dividing and persisted cells, supporting its application in persistent and chronic infections (Defraine et al., 2016).

#### 4.2.2 Therapeutic optimization and translational potential

Synergistic interactions further strengthen therapeutic applicability, as eAb Endolysin enhances *β*-lactam efficacy (Islam et al., 2022). Because endolysins target peptidoglycan, a structural component not directly affected by most conventional antibiotics that primarily interfere with protein synthesis, DNA replication, or metabolic pathways, their mechanism reduces cross-resistance potential and supports application against MDR strains (Lim et al., 2022), with DS-PA90 showing sustained activity and minimal resistance development *in vitro* and Lys22 demonstrating broad-spectrum biofilm activity alongside downregulation of virulence-associated genes (Yang et al., 2025).

Additional candidates, such as LysAB54 and LysAB1245 exhibit rapid bactericidal effects, stability across varying environmental conditions, and effectiveness across multiple capsular types, reinforcing the translational promise of engineered lysins as direct lytic agents within advanced antimicrobial strategies (Khan et al., 2021; Soontarach et al., 2024).

Phage therapy offers several intrinsic advantages that distinguish it from conventional antimicrobial strategies. High specificity enables selective targeting of pathogenic bacteria without disrupting beneficial microbiota, thereby minimizing collateral damage and microbiota-associated complications. The capacity to penetrate and dismantle biofilms enhances efficacy against chronic and device-associated infections (Figure 2). Moreover, the self-amplifying nature of phages allows replication at the site of infection, increasing local phage concentration in response to bacterial density and sustaining antibacterial pressure during active infection (Chan et al., 2013; Kortright et al., 2019; Principi et al., 2019).

### 4.3 Monoclonal antibodies and targeted immunotherapy

#### 4.3.1 Antibody targets and protective mechanisms

Monoclonal antibodies (mAbs) can specifically recognize bacterial surface antigens, including capsular polysaccharides, outer membrane proteins, and lipopolysaccharides, thereby promoting opsono-phagocytic clearance, complement activation, and neutralization of virulence factors (McConnell, 2019; Nielsen et al., 2021; Slarve et al., 2023). Enhancement of opsono-phagocytosis constitutes a central mechanism of antibody-mediated protection against *A. baumannii*, whereby monoclonal antibodies promote immune recognition, bacterial clearance, and improved survival outcomes.

Monoclonal antibodies such as mAb 8E6 and mAb 1B5 bind surface-expressed ATP synthase, significantly increasing opsonization and facilitating phagocytic uptake (Figure 2), resulting in enhanced bacterial clearance and reduced disease severity in murine respiratory infection models (Zeng et al., 2020). Similarly, capsule-directed antibodies including MAb C8 and MAb 65 bind capsular polysaccharides, overcome capsule-mediated immune evasion, enhance macrophage-mediated opsono-phagocytosis, reduce bacterial burden and bloodstream density, and improve survival in infection and sepsis models, while MAb 65 additionally demonstrates synergy with colistin and modulates cytokine responses to optimize therapeutic outcomes. The development of antibodies targeting multiple capsular types broadens coverage against genetically diverse clinical isolates and addresses capsular heterogeneity (Nielsen et al., 2021).

Complement activation is another major protective pathway, as antibodies targeting capsular polysaccharides and O-antigen carbohydrates, such as MAb5, trigger complement-mediated lysis and promote C3b and iC3b deposition (Figure 2), thereby enhancing neutrophil-mediated phagocytosis, although complement sensitivity varies according to capsular composition and high-molecular-weight mucoid phenotypes exhibit reduced C3b deposition and increased resistance (Kamuyu et al., 2021; Gong et al., 2022; Slarve et al., 2023).

Certain mAbs exert direct bactericidal activity independent of host effector mechanisms; Pse-MAB1, targeting pseud aminic acid, confers protection in murine infection models, highlighting therapeutic potential in immunocompromised settings (Yang et al., 2024). While single-chain variable fragment antibodies such as EB211 and EB279 demonstrate strong bacterial binding and significant reductions in viability, with enhanced efficacy when combined with last-resort antibiotics (Basardeh et al., 2022).

Outer membrane proteins (OMPs) serve as accessible and conserved antibody targets; fully human mAbs against Omp38 inhibit adherence and biofilm formation across diverse strains (Zhang et al., 2025). Antibodies directed against OmpA, a key virulence factor mediating adhesion, biofilm formation, immune evasion, and host cell apoptosis, reduce bacterial adherence, internalization, and proliferation in alveolar epithelial cells. They also enhance macrophage-mediated killing, diminish biofilm formation, and improve host cell survival (Yeganeh et al., 2022; Barati et al., 2024). In addition OmpA-derived peptides have demonstrated immunogenicity in exposed healthcare workers, supporting vaccine relevance (Paydarfar et al., 2023).

LPS and lipooligosaccharides (LOS) are important surface components that help maintain membrane integrity and support immune evasion. Structural changes in LPS/LOS can reduce bacterial susceptibility to phage infection and complement-mediated killing. However, antibodies targeting LPS/LOS can interfere with these protective adaptations and promote immune-mediated bacterial clearance (Moffatt et al., 2013; Kamoshida et al., 2020; Akoolo et al., 2022), In addition, other surface-associated targets, such as OXA-23, have been identified using human immune repertoire transgenic mice, showing potential for prophylactic protection against carbapenem-resistant strains (Baker et al., 2024).

#### 4.3.2 Broad-spectrum and vaccine-based immunotherapeutic approaches

Broad-spectrum monoclonal antibodies, including MAb5 and MAb65, show protective activity against multiple clinical isolates of *A. baumannii*. MAb5 primarily acts by binding surface carbohydrate antigens and activating the complement pathway (Figure 2), which promotes C3b/iC3b deposition and enhances neutrophil-mediated phagocytosis. In contrast, MAb65 targets capsular polysaccharides and improves macrophage-mediated opsono-phagocytosis, thereby reducing bacterial burden and improving survival in animal infection models. MAb65 has also shown synergistic activity with colistin, suggesting its potential use as an adjunctive therapy. Because capsular antigens are highly variable among clinical isolates, antibodies directed against more conserved protein antigens may provide broader cross-protection. In addition, multi-strain immunization strategies can induce wider antibody responses and improve protection against genetically diverse *A. baumannii* isolates (Kamuyu et al., 2021; Nielsen et al., 2021; Slarve et al., 2023).

Overall, antibody-based immunotherapy can be designed to protect against *A. baumannii* through several mechanisms, including enhanced opsonization, complement activation, and inhibition of bacterial attachment. These approaches support both prophylactic and therapeutic applications (Emami et al., 2023), Vaccine-based strategies also highlight the importance of humoral immunity against *A. baumannii*. For example, the Acinetobacter Multi-Epitope Vaccine (AMEV2) induces high antibody titres, promotes macrophage-mediated bacterial killing, and protects animals against lethal infection (Jeffreys et al., 2022), Similarly, whole-cell vaccine candidates generate protective antibody responses that can be transferred through passive serum administration (Dollery et al., 2022). Egg yolk-derived immunoglobulins (IgYs) targeting the FimA virulence factor protect against pneumonia in murine models (Shaygankho et al., 2023). In addition, passive transfer of immune serum from animals immunized with the Abp2D adhesin reduces bacterial colonization in catheter-associated urinary tract infection models, supporting serum-based immunoprophylaxis for high-risk clinical settings (Timm et al., 2025).

### 4.4 CRISPR-based strategies

#### 4.4.1 CRISPR-Cas systems as programmable antimicrobial tools

Clustered regularly interspaced short palindromic repeats (CRISPR) and CRISPR-associated (Cas) systems have emerged as powerful genome-editing and antimicrobial technologies for combating MDR *A.baumannii*. These RNA-guided nucleases enable sequence-specific targeting of bacterial DNA, allowing selective disruption of antimicrobial resistance genes, elimination of resistance-carrying plasmids (Figure 2), and precise genome editing, thereby offering a programmable strategy for antimicrobial intervention and molecular diagnostics (Bikard et al., 2014; Pursey et al., 2018; Allemailem, 2024).

Within the ABC complex, diverse CRISPR-Cas subtypes, including I-Fa, I-Fb, and I-Fv, are present in approximately 26.3% of analyzed genomes and are frequently associated with toxin–antitoxin systems and prophage regions, reflecting their role in genome defense and plasticity (Mancilla-Rojano et al., 2020). The native Type I-F system has been successfully harnessed for genome editing and transcriptional repression, enabling efficient gene knockout and regulation of resistance determinants (Yao et al., 2023).

Among engineered platforms, CRISPR-Cas9 has been extensively applied to selectively disrupt resistance loci through guide RNA–directed double-strand DNA cleavage, targeting clinically significant genes such as *blaNDM-1*, *mcr-1*, and *blaOXA-23-like* (Figure 2), thereby limiting carbapenem and colistin resistance and resensitizing resistant strains to antibiotics (Junaid et al., 2023; Allemailem, 2024; Gurung et al., 2024). The CRISPR-FnCpf1 system further expands genomic targeting capacity by enabling large-fragment deletions and multiplex gene editing, facilitating simultaneous modification of multiple loci and enhancing plasmid-curing strategies.

#### 4.4.2 Delivery challenges and combination strategies

To reduce the emergence of escape mutants, integrated approaches such as the AssociaTes Toxin–Antitoxin with CRISPR-Cas to Kill (ATTACK) strategy combine CRISPR-mediated targeting with toxin–antitoxin systems to ensure bacterial elimination even if CRISPR activity is compromised (Zhu, 2023; Wang et al., 2023).

Successful clinical translation depends on efficient delivery systems, with engineered bacteriophages, conjugative plasmids, and nanoparticle-based carriers (Figure 2) being investigated to facilitate intracellular delivery of CRISPR components (Mayorga-Ramos et al., 2023; Zhu, 2023; Allemailem, 2024). However, potential resistance mechanisms such as anti-CRISPR proteins and genomic rearrangements, along with the need for optimized guide RNA design to minimize off-target effects, remain important considerations (Yao et al., 2023; Zhu, 2023).

Integration of CRISPR platforms with complementary antimicrobial approaches may improve therapeutic efficacy, including antimicrobial peptides and bacteriophage therapy, offers a multifaceted strategy to enhance antimicrobial efficacy and address persistent resistance in *A. baumannii* (Bikard et al., 2014; Pursey et al., 2018).

## 5 Conclusion

The *Acinetobacter baumannii–calcoaceticus* complex remains one of the most formidable groups of nosocomial pathogens because of its marked genomic plasticity, capacity to accumulate diverse resistance determinants, and persistence in hospital environments. Although major advances have been made in understanding the molecular basis of resistance, clinical management continues to be hindered by the close relatedness of species within the complex and by the rapid global dissemination of multidrug-resistant and carbapenem-resistant lineages. Accurate species-level identification is therefore essential not only for taxonomy and epidemiologic surveillance, but also for improving interpretation of resistance patterns, outbreak investigation, and therapeutic decision-making. However, routine phenotypic and biochemical systems still lack sufficient discriminatory power for reliable differentiation within the complex, and even MALDI-TOF MS performance remains dependent on database completeness and curation. Whole-genome sequencing offers the highest resolution for species assignment, typing, and resistance prediction, but its routine implementation is still constrained in many settings by cost, infrastructure requirements, turnaround time, and the need for specialized bioinformatic expertise.

Therapeutically, the ABC complex continues to present a major challenge because conventional options are limited by extensive resistance, suboptimal efficacy, and drug-related toxicity. Recent progress with sulbactam–durlobactam is an important advance, and current IDSA guidance recognizes sulbactam-based therapy as central to the treatment of carbapenem-resistant *A. baumannii*, supported by phase 3 clinical trial data showing improved safety relative to colistin-based regimens. Nevertheless, treatment success remains threatened by the emergence of additional resistance mechanisms, hetero resistance, limited access to newer agents in many regions, and the continued need for combination therapy in severe infections. Innovative approaches such as bacteriophage therapy, engineered endolysins, monoclonal antibodies, and CRISPR-based antimicrobials are highly promising, but at present they remain constrained by narrow host range, delivery barriers, manufacturing and regulatory challenges, and the relative scarcity of robust clinical efficacy data. Taken together, progress against the ABC complex will require an integrated strategy that combines accurate and accessible diagnostics, genomic surveillance, antimicrobial stewardship, infection-prevention measures, and carefully validated pathogen-directed therapeutics.
